# Definition of malnutrition from routinely-collected data for orthopedic surgery research: the global leadership initiative on malnutrition (GLIM) tool and others

**DOI:** 10.3389/fnut.2023.1200049

**Published:** 2023-11-09

**Authors:** Matteo Briguglio, Thomas W. Wainwright, Giovanni Lombardi

**Affiliations:** ^1^Laboratory of Nutritional Sciences, IRCCS Orthopedic Institute Galeazzi, Milan, Italy; ^2^Orthopaedic Research Institute, Bournemouth University, Bournemouth, United Kingdom; ^3^University Hospitals Dorset, NHS Foundation Trust, Poole, United Kingdom; ^4^Laboratory of Experimental Biochemistry and Molecular Biology, IRCCS Orthopedic Institute Galeazzi, Milan, Italy; ^5^Department of Athletics, Strength and Conditioning, Poznań University of Physical Education, Poznań, Poland

**Keywords:** orthopaedics, surgery, malnutrition, prehabilitation, enhanced recovery after surgery, health status, patient outcomes assessment, blood chemical analyses

## Abstract

The correct identification of malnourished patients in the context of hip, knee, or spine surgery research would enhance the quality of analytical studies investigating the prediction potential of preoperative nutritional disorders on postoperative recovery. However, accurate malnutrition screening and diagnostic assessment rely on parameters that were not routinely collected in routine practice until a few years ago. The authors of this article present substitute literature-based equations that can be built up using historical routinely collected data to classify patients that had been at risk of malnutrition or malnourished. For what concerns the risk screening, several methods are available to identify patients at risk of over- or undernutrition, encompassing the BWd (body weight difference from the ideal weight), GNRI (geriatric nutritional risk index), INA (instant nutritional assessment), LxA (combination of lymphocyte count and albumin), PMA (protein malnutrition with acute inflammation), PMAC (protein malnutrition with acute and chronic inflammation), IDM (iron deficit malnutrition), and VBD (vitamin B deficit malnutrition). Conversely, the GLIM (global leadership initiative on malnutrition) criteria can be used to assess malnutrition and diagnose subclasses of undernutrition. Rational use of these tools can facilitate the conduction of efficient prospective studies in the future, as well as bespoke retrospective cohort studies and database research.

## 1. We need tools to identify malnutrition in retrospective research studies

Malnutrition is a polyhedric condition whose etiology lies in the failure of the individual to meet the nutritional requirements, reduced or excessive food intake, or an unspecified alteration of the nutritional status from ailments or medications. As many as one in two patients undergoing joint or spine surgery is at risk of malnutrition, is malnourished, or will be after surgery (1–3). Malnutrition causes profound changes to the host’s anatome and physiome, undermining daily activities and resilience to cope with distressing events (4). Major orthopedic surgery initiates a surgical stress response, exposing malnourished patients to a greater risk of complications and slow/impaired recovery (5). The correction of malnutrition is proposed to be one of the key elements of prehabilitation in orthopedic surgery, with dietary interventions playing an important role in optimizing the nutritional status, preventing adverse events, and enhancing recovery (6). Nutrition screening relies on quick and validated screening tool, such as the malnutrition screening tool (MST) (7), nutritional risk screening-2002 (NRS-2002) (8), or the malnutrition universal screening tool (MUST) (9), which all investigate the risk of being malnourished according to patients’ replies. The diagnosis of malnutrition is achieved using the framework formulated by the global leadership initiative on malnutrition (GLIM) (10). This assessment, diagnosis, and grading scheme is performed in patients at risk of malnutrition, considering the presence of non-volitional weight loss, hypophagia, abnormal body composition, muscular weakness, and the disease state. The finding of these signs requires trained personnel and devices for body analysis and testing, which were not routinely performed until recently. Consequently, there exists a vast amount of historical data that lacks the necessary information to explore the incidence and role of malnutrition in orthopedic surgery research.

In this article, we present literature-based indicators of nutritional disorders (undernutrition, overnutrition, and micronutrient abnormalities) that can be calculated from variables commonly part of most orthopedic centers’ clinical practice. These equations can be used prospectively and in the context of bespoke retrospective cohort studies and database research on joint arthroplasty and spine surgery. Nutrition-related conditions like cachexia, sarcopenia, and frailty are not debated in this perspective.

## 2. Equations to calculate the risk of malnutrition from routinely-collected data

The risk of malnutrition can be inferred through equations shown in Table 1. First, we propose the calculation of the body weight difference (BWd) (11) from the ideal body weight (IBW), which informs the clinician how much the actual body weight (ABW) of the patient deviates from the reference value. If the difference is clinically relevant (usually identified as greater than 10%) then the subject may be considered at risk of undernutrition or overnutrition although it is not possible to know in retrospect whether the difference in weight is due to a non-volitional loss or gain. Second is the geriatric nutritional risk index (GNRI) (12), which combines one of the most used nutritional analytes (albumin) with a consideration of the patient’s weight similar to the BWd method. Third is the instant nutritional assessment (INA) (13), which was formulated in the second half of the nineties but is still relevant since it combines albumin (ALB) with total lymphocytic count (LYMPC), both being well-known indicators of nutritional status. Similarly, the numerical product of the two analytes (LxA) is the fourth nutrition-related score that we suggest for patient grouping (14). The fifth score defines patients at risk of protein malnutrition with acute inflammation (PMA) based on low ALB and high CRP (15) and, similarly, there is the sixth score that helps identify patients at risk of malnutrition based on elevated markers of acute/chronic inflammation and low proteins (PMAC). This latter has been adapted by the authors from the prognostic inflammatory and nutritional index (16), which contrariwise included the α1-acid glycoprotein as a second acute-phase reactant other than the CRP. The last two calculations that are proposed should theoretically signify the presence of certain specific nutritional deficits, being the deficit of iron (IDM) (17) or B vitamins (VBD) (18).

**Table 1:** Calculations built up with routinely-collected parameters to identify malnutrition in orthopedic surgery patients.

Score	Equations	Notes and formulation
	To define patients with a potential diagnosis of malnutrition (undernutrition)	
GLIM	Clean undernutrition: BMI $\left(\frac{\mathrm{kg}}{m^{2}}\right)$ < 20 if adults OR < 22 if seniors AND ↓ CRP AND ↓ NLR AND ↓ ASAPS DRM with inflammation: [BMI $\left(\frac{\mathrm{kg}}{m^{2}}\right)$ < 20 if adults OR < 22 if seniors] AND ↑ ASAPS AND [ ↑ CRP OR ↑ NLR] DRM without inflammation: [BMI $\left(\frac{\mathrm{kg}}{m^{2}}\right)$ < 20 if adults OR < 22 if seniors] AND ↑ ASAPS AND [ ↓ CRP OR ↓ NLR]	GLIM^a^ was based on a global consensus (32). It requires at least one phenotypic (low BMI) AND one etiologic (disease/inflammation) criterion. The authors propose the use of ASAPS is ≥2, CRP > 5 mg·L-1, and NLR ≥ 6.
	To define patients potentially at risk of malnutrition	
BWd	Risk of undernutrition based on a MCID weight loss: [ABW − IBW $\left(\mathrm{kg}\right)$ ] < 10% IBW $\left(\mathrm{kg}\right)$ Risk of overnutrition based on a MCID weight gain: [ABW $\left(\mathrm{kg}\right)$ − IBW $\left(\mathrm{kg}\right)$ ] > 10% IBW $\left(\mathrm{kg}\right)$	BWd^b^ is proposed by the authors of this article based on the MCID for weight change (11), being a loss or gain greater than 10% IBW.
GNRI	Risk of malnutrition based on weight loss and proteins: [1.489 × ALB $\left(\frac{g}{L}\right)$ ] + [41.7 × ABW $\left(\mathrm{kg}\right)$ ÷ IBW $\left(\mathrm{kg}\right)$ ]^c^	GNRI was based on the risk of adverse outcomes in a cohort of older patients admitted to a geriatric rehabilitation care unit (12).
INA	Risk of energy malnutrition: ALB ≥ 3.5 $\left(\frac{g}{\mathrm{dL}}\right)$ AND LYMC < 1,500 $\left(\frac{\mathrm{cells}}{\mathrm{\mu L}}\right)$ Risk of protein malnutrition: ALB < 3.5 $\left(\frac{g}{\mathrm{dL}}\right)$ AND LYMC ≥ 1,500 $\left(\frac{\mathrm{cells}}{\mathrm{\mu L}}\right)$ Risk of protein-energy malnutrition: ALB < 3.5 $\left(\frac{g}{\mathrm{dL}}\right)$ AND LYMC < 1,500 $\left(\frac{\mathrm{cells}}{\mathrm{\mu L}}\right)$	INA was based on the risk of adverse outcomes in a cohort of patients admitted to a multispecialty hospital (13).
LxA	Risk of malnutrition based on immune function and proteins: LYMC $\left(\frac{\mathrm{cells}}{\mathrm{\mu L}}\right)$ × ALB $\left(\frac{g}{\mathrm{dL}}\right)$	LxA was based on the risk of adverse outcomes in a cohort of patients with stage II/III rectal cancer (14).
PMA	Risk of malnutrition based on markers of acute inflammation and proteins: CRP $\left(\frac{\mathrm{mg}}{L}\right)$ ÷ ALB $\left(\frac{g}{\mathrm{dL}}\right)$	PMA was based on the risk of adverse outcomes in a cohort of critically ill patients in an acute medical ward (15).
PMAC	Risk of malnutrition based on markers of acute/chronic inflammation and proteins: [NLR + CRP $\left(\frac{\mathrm{mg}}{L}\right)$ ] ÷ [ALB $\left(\frac{g}{\mathrm{dL}}\right)$ + PALB $\left(\frac{\mathrm{mg}}{L}\right)$ ]	The PMAC is proposed by the authors of this article. It has been adapted from the prognostic inflammatory and nutritional index (16).
IDM	risk of malnutrition based on body weight and markers of iron homeostasis: ABW $\left(\mathrm{kg}\right)$ × [IHB^d^ $\left(\frac{g}{L}\right)$ − AHB $\left(\frac{g}{L}\right)$ ] × 2.4 + 500 mg	The Ganzoni equation is ideally used to select appropriate iron deficit repletion dosing in patients with iron deficit anemia (17).
VBD	risk of malnutrition based on markers of iron homeostasis: ↑ MCV AND ↑ MCH OR ↓ MCHC	The VBD is proposed by the authors of this article based on the risk of adverse outcome in orthopedic patients with macrocytic hyperchromic anemia (25).

^a^Low BMI for GLIM criteria is < 20 kg/m^2^ if subjects are < 70 years old or < 22 kg/m^2^ if ≥ 70 years old. Based on these thresholds, the authors of this article derived a BMI categorization for patients from 18 to 70 years of age: < 20.0 kg/m^2^ (underweight), 20.0–26.4 kg/m^2^ (normal), 26.5–31.4 kg/m^2^ (overweight), 31.5–36.4 kg/m^2^ (obesity I), 36.5–41.4 kg/m^2^ (obesity II), ≥ 41.5 kg/m^2^ (obesity III). Similarly, the inferred BMI categorization for patients ≥ 70 years of age is: < 22.0 kg/m^2^ (underweight), 22.0–28.4 kg/m^2^ (normal), 28.5–33.4 kg/m^2^ (overweight), 33.5–38.4 kg/m^2^ (obesity I), 38.5–43.4 kg/m^2^ (obesity II), ≥ 43.5 kg/m^2^ (obesity III). ^b^The BWd is the difference between the patient’s ABW and the IBW (assumed for a person of the same gender), with a percentage difference over 10% being considered MCID. The IBW in the BWd formula is calculated using the Devine (33) equations, being in men = 50 kg + 2.3 kg × (height, inches − 60) and in women = 45.5 kg + 2.3 kg × (height, inches − 60). ^c^The ration between ABW and IBW is set as equal to 1 if ABW is higher than IBW. The IBW in the GNRI formula is calculated using the Lorentz (12) equations, being in men = (height, cm) −100 − [(height, cm − 150) ÷ 4] and in women = (height, cm) − 100 − [(height, cm − 150) ÷ 2.5]. ^d^IHB is the mean value of the normal reference range, which can be 15.6 g·dL-1 for males and 13.5 g·dL-1 for females. GLIM, global leadership initiative on malnutrition; BMI, body mass index; CRP, C-reactive protein; NLR, neutrophil-lymphocyte ratio; ASAPS, American society of anesthesiologists classification of physical status; DRM, disease-related malnutrition (undernutrition); BWd, body weight difference; MCID, minimal clinically important difference; ABW, actual body weight; IBW, ideal body weight; GNRI, geriatric nutritional risk index; ALB, albumin; INA, instant nutritional assessment; LYMC, lymphocytes count; LxA, combination of lymphocyte count and albumin; PMA, protein malnutrition with acute inflammation; PMAC, protein malnutrition with acute and chronic inflammation; PALB, transthyretin or prealbumin; IDM, iron deficit malnutrition; IHB, ideal hemoglobin; AHB, actual hemoglobin; VBD, vitamin B deficit; MCV, mean corpuscular volume; MCH, mean corpuscular hemoglobin; MCHC, mean corpuscular hemoglobin concentration.

## 3. The GLIM equation from routinely-collected data to diagnose malnutrition

Even if the information necessary to apply the canonical GLIM diagnostic scheme is not available among the historical data, it is possible to infer a probable diagnosis of malnutrition using the substitute literature-based equation shown in Table 1. GLIM versatility has allowed its application in various clinical settings and study designs, and it has already been used in orthopedic surgery research (1, 2, 19) as an alternative framework to the classical diagnostic process (20). The GLIM equation characterizes patients according to different combinations of phenotypic (percentage of unintentional weight loss since last evaluation, low body mass index, reduced muscle mass) and etiological (reduced food intake or assimilation, inflammation, disease burden) criteria. In orthopedic surgery research, the phenotypic criterion accessible from clinical practice is often the body mass index (BMI), while diverse etiological criteria can be selected among different markers and indexes. We propose the use of the American society of anesthesiologists classification of physical status (ASAPS), C-reactive protein (CRP), and neutrophil-lymphocyte ratio (NLR) to discriminate clean malnutrition, disease-related malnutrition (DRM) with inflammation, and DRM without inflammation. The ASAPS is an indicator of disease burden and follows the next categorical coding: healthy = 1, mild disease = 2, severe disease = 3, threat to life = 4, moribund = 5, brain-dead = 6. The use of GLIM requires the sample study to be classified according to precise age ranges (< 40 years = younger adults; 40–70 years = adults; ≥ 70 years = older adults) and BMI categories that identify underweight as different than usual (< 20.0 kg/m^2^ if age < 70 years or < 22.0 kg/m^2^ if age ≥ 70 years). Although the BMI alone has not been offered in this article as an indicator of the nutritional status for its already acclaimed practice, it is important to highlight that its use according to the traditional labeling is archaic, especially in old patients whose height is profoundly changed and the aging process parallels with a shift in the health risk given by body compositional changes (21). Therefore, if it really were to be used, it would be worth correcting the Quetelet index (22) in agreement with the more recent knowledge on the protective role of fat. Consequently, in younger adults and adults, BMI < 18.5 kg/m^2^ is underweight, 18.5–24.9 kg/m^2^ is normal, 25.0–29.9 kg/m^2^ is overweight, 30.0–34.9 kg/m^2^ is obesity I, 35.0–39.9 kg/m^2^ is obesity II, and ≥ 40.0 kg/m^2^ is obesity III. In seniors, it might be appropriate to consider that BMI < 25.0 kg/m^2^ is underweight, 25.0–35.0 kg/m^2^ is normal, 35.1–40.0 kg/m^2^ is overweight, 40.1–45.0 kg/m^2^ is obesity I, 45.1–50.0 kg/m^2^ is obesity II, and ≥ 50.1 kg/m^2^ is obesity III (23). Concerning the CRP and NLR, their circulating levels are considered representative of acute and chronic inflammation, respectively, with the latter more accurately being able to differentiate a state of severe (≥ 8), moderate (6–7.99), mild (4–5.99), low (2–3.99), or normal (< 2) chronic inflammatory status.

## 4. What is the value of using these scores?

The incredible amount of historical data that lacks the information necessary for a correct diagnosis can nonetheless count on a set of non-diagnostic indicators each with its own drawbacks (Table 2) but having in common the fact that they couple at least two surrogate variables of nutritional interest. It is not meaningless to use a single laboratory analyte as an indicator of nutritional status, since they have long been used by themselves (especially albumin and hemoglobin) (21), and still provide remarkable findings in today’s orthopedic surgery research (24, 25). However, it is necessary to distinguish that risk screening and diagnosis are two distinct evaluations but part of the same two-step process. Therefore, the authors recommend the selection of at least one screening tool among those presented in this article together with the semi-gold standard GLIM when exploring the prevalence of malnutrition or its risk or the prediction potential on orthopedic surgery outcome in retrospective analytical studies. The concurrent use of machine learning techniques is also advised to further explore the actual weight of each anthropometric, biochemical, and disease-related variable used.

**Table 2:** Characteristics of the scores, suggested coding system, and limitations.

Score	Nature	Units	Categorical coding	Limitations
GLIM	Nominal Ordinal	None	Clean undernutrition = 1 Disease-related undernutrition without inflammation = 2 Disease-related undernutrition with inflammation = 3	Non applicable in adult patients with BMI ≥ 20 and seniors with BMI ≥ 22.
BWd	Continuous	kg	No risk of malnutrition = 0 Risk of undernutrition (gain greater than 10% IBW) = 1 Risk of overnutrition (loss greater than 10% IBW) = 2	Non applicable in patients that have the absolute difference between ABW and IBW within 10% IBW. Non applicable in patients without ABW and height.
GNRI	Continuous	None	No risk of malnutrition = 0 Low risk of malnutrition = 1 Moderate risk of malnutrition = 2 Major risk of malnutrition = 3	Non applicable in younger adult and adult patients. Non applicable in patients without ALB, ABW, and height. Categories of nutrition-related risk are major (< 82), moderate (82–91.9), low (92–98), and no risk (> 98).
INA	Nominal Ordinal	None	No risk of malnutrition = 0 Risk of protein malnutrition = 1 Risk of energy malnutrition = 2 Risk of protein-energy malnutrition = 3	Non applicable in patients without ALB or LYMPC. score classifies patients into groups of nutrition-related risk according to circulating levels of albumin and lymphocytes.
LxA	Continuous	None	No risk of malnutrition = 0 Moderate risk of malnutrition = 1 High risk of malnutrition = 2	Non applicable in patients without LYMPC or ALB. Categories or nutrition-related risk: poor (≤ 4,515), middle (4515–7,920), good nutrition (> 7,920).
PMA	Continuous	None	No risk = 0 Low risk = 1 Moderate risk = 2 High risk = 3	Non applicable in patients without CRP or ALB. Categories of nutrition-related risk: no risk (< 0.4), low risk (0.4–1.2), moderate risk (1.2–2.0), high risk (≥ 2.0).
PMAC	Continuous	None	Cohort-based percentiles: < 25th = 0; ≥ 25th and < 50th = 1; ≥ 50th and < 75th = 2; ≥ 75th = 3	Non applicable in patients without CRP, NLR, ALB, or PALB. Categories of nutrition-related risk: < 25th percentile, ≥ 25th percentile and < 50th percentile, ≥ 50th percentile and < 75th percentile; ≥ 75th percentile.
IDM	Continuous	mg	Cohort-based percentiles: < 25th = 0; ≥ 25th and < 50th = 1; ≥ 50th and < 75th = 2; ≥ 75th = 3	Non applicable in patients with IHB lower than AHB. Categories of nutrition-related risk: < 25th percentile, ≥ 25th percentile and < 50th percentile, ≥ 50th percentile and < 75th percentile; ≥ 75th percentile.
VBD	Nominal Dichotomous	None	Adequate vitamin B status = 0 Functional vitamin B deficiency = 1	Non applicable in patients without MCV, MCH or MCHC the VBD categorizes patients in at risk and not at risk.

GLIM, global leadership initiative on malnutrition; DRM, disease-related malnutrition; BMI, body mass index; BWd, body weight difference; ABW, actual body weight; IBW, ideal body weight; GNRI, geriatric nutritional risk index; ALB, albumin; INA, instant nutritional assessment; LxA, combination of lymphocyte count and albumin; PMA, protein malnutrition with acute inflammation; CRP, C-reactive protein; PMAC, protein malnutrition with acute and chronic inflammation; NLR, neutrophil-lymphocyte ration; PALB, prealbumin; IDM, iron deficit malnutrition; IHB, ideal hemoglobin; VBM, vitamin B deficit malnutrition.

Concerning current clinical practice, the integration of valid and short tools, such as the four questions of the nutritional risk screening-2002 (NRS-2002-4Q) (26), the mini nutritional assessment-short form (MNA-SF) (27), or the patient generated-subjective global assessment short form (PG-SGA SF) (28) may be a valuable intermediate step to screen malnutrition in orthopedic surgery pathways. However, the much more valuable diagnostic frameworks that combine dietetic, anthropometric, biochemical, and functional variables like the GLIM (29) ought to be systematically incorporated as soon as feasible because of its cost-effectiveness (6, 18, 30).

## 5. Final considerations

Different criteria based on routinely collected data can be used to determine the prevalence of patients at risk of being malnourished or those suffering from malnutrition in the context of hip, knee, or spine surgery outcomes. When analyzing historical data, patients at risk of undernutrition can be identified using several equations, including the BWd based on the MCID weight loss, the GNRI, different combinations of laboratory parameters (INA, LxA, PMA, PMAC), and the IDM or VBD that determine the risk that the patient may suffer from an iron deficit or macrocytic hyperchromic anemia, respectively. Equally, the GLIM equation ought to be considered the reference calculation to diagnose undernutrition, while for the identification of overnutrition, we argue that the BWd calculation based on the MCID weight gain can be used. Our proposed literature-based equations come with flaws, being mere substitutes for the definition of the risk of being malnourished or the diagnosis of malnutrition. The lack of information regarding unintentional weight loss, muscle mass, food intake, and absorption might potentially misjudge the real prevalence of nutritional disorders. However, reported in tandem (31), a rational and cautious use of these tools will shed the light on the role of an unbalanced nutritional status in orthopedic patients and facilitate the conduction of prospective studies, bespoke retrospective cohort studies, and database research probing risk factors or prediction models.

## Data availability statement

The original contributions presented in the study are included in the article/supplementary material, further inquiries can be directed to the corresponding author.

## Author contributions

MB formulated the first draft. TW and GL revised and integrated the manuscript. All authors agreed to be accountable for the content of the work and submitted the final version to this manuscript.
